# Deep learning to automate the labelling of head MRI datasets for computer vision applications

**DOI:** 10.1007/s00330-021-08132-0

**Published:** 2021-07-20

**Authors:** David A. Wood, Sina Kafiabadi, Aisha Al Busaidi, Emily L. Guilhem, Jeremy Lynch, Matthew K. Townend, Antanas Montvila, Martin Kiik, Juveria Siddiqui, Naveen Gadapa, Matthew D. Benger, Asif Mazumder, Gareth Barker, Sebastian Ourselin, James H. Cole, Thomas C. Booth

**Affiliations:** 1 School of Biomedical Engineering & Imaging Sciences, Kings College London, Rayne Institute, 4th Floor, Lambeth Wing, London, SE1 7EH UK; 2 Department of Neuroradiology, Ruskin Wing, King’s College Hospital NHS Foundation Trust, London, SE5 9RS UK; 3 Wrightington, Wigan & Leigh NHSFT, Wigan, WN1 2NN UK; 4 Hospital of Lithuanian University of Health Sciences, Kaunas Clinics, Kaunas, Lithuania; 5 Department of Neurology, Ruskin Wing, King’s College Hospital NHS Foundation Trust, London, SE5 9RS UK; 6 Guy’s and St Thomas’ NHS Foundation Trust, Westminster Bridge Road, London, SE1 7EH UK; 7 Institute of Psychiatry, Psychology & Neuroscience, King’s College London, London, SE5 8AF UK; 8 Centre for Medical Image Computing, Department of Computer Science, University College London, London, WC1V 6LJ UK; 9 Dementia Research Centre, University College London, London, WC1N 3BG UK

**Keywords:** Deep learning, Natural language processing, Magnetic resonance imaging, Data curation, Radiology

## Abstract

**Objectives:**

The purpose of this study was to build a deep learning model to derive labels from neuroradiology reports and assign these to the corresponding examinations, overcoming a bottleneck to computer vision model development.

**Methods:**

Reference-standard labels were generated by a team of neuroradiologists for model training and evaluation. Three thousand examinations were labelled for the presence or absence of any abnormality by manually scrutinising the corresponding radiology reports (‘reference-standard report labels’); a subset of these examinations (*n* = 250) were assigned ‘reference-standard image labels’ by interrogating the actual images. Separately, 2000 reports were labelled for the presence or absence of 7 specialised categories of abnormality (acute stroke, mass, atrophy, vascular abnormality, small vessel disease, white matter inflammation, encephalomalacia), with a subset of these examinations (*n = *700) also assigned reference-standard image labels. A deep learning model was trained using labelled reports and validated in two ways: comparing predicted labels to (i) reference-standard report labels and (ii) reference-standard image labels. The area under the receiver operating characteristic curve (AUC-ROC) was used to quantify model performance. Accuracy, sensitivity, specificity, and F1 score were also calculated.

**Results:**

Accurate classification (AUC-ROC > 0.95) was achieved for all categories when tested against reference-standard report labels. A drop in performance (ΔAUC-ROC > 0.02) was seen for three categories (atrophy, encephalomalacia, vascular) when tested against reference-standard image labels, highlighting discrepancies in the original reports. Once trained, the model assigned labels to 121,556 examinations in under 30 min.

**Conclusions:**

Our model accurately classifies head MRI examinations, enabling automated dataset labelling for downstream computer vision applications.

**Key Points:**

• *Deep learning is poised to revolutionise image recognition tasks in radiology; however, a barrier to clinical adoption is the difficulty of obtaining large labelled datasets for model training.*

• *We demonstrate a deep learning model which can derive labels from neuroradiology reports and assign these to the corresponding examinations at scale, facilitating the development of downstream computer vision models.*

• *We rigorously tested our model by comparing labels predicted on the basis of neuroradiology reports with two sets of reference-standard labels: (1) labels derived by manually scrutinising each radiology report and (2) labels derived by interrogating the actual images.*

**Supplementary Information:**

The online version contains supplementary material available at 10.1007/s00330-021-08132-0.

## Introduction

Deep learning computer vision systems are poised to revolutionise image recognition tasks in radiology [1–3]. However, progress has been constrained by a critical bottleneck; during training, artificial neural networks often require tens of thousands of labelled images to achieve the best possible performance. Unlike traditional computer vision tasks, where image annotation is simple (e.g. labelling cat, dog, horse) and large-scale labelling can be crowdsourced [4], assigning radiological labels is highly complex, requiring considerable domain expertise. Manually labelling MRI scans appears to be particularly laborious due to (1) the superior soft-tissue contrast of MRI which enables more refined diagnoses compared with other imaging modalities such as computed tomography; and (2) the use of multiple, complementary imaging sequences so that a larger number of images must be scrutinised per examination. Given the year-on-year increase in MRI scan demand for at least a decade [5] and the existing pressures on clinical services seen in many countries, it also appears to be particularly difficult to justify using radiologists’ time to generate labelled MRI datasets for research purposes. As a result, it is plausible that neuroradiology, where MRI is fundamental, is at risk of not being able to fully harness deep learning computer vision methodology for image recognition tasks.

A promising alternative to manual dataset labelling is to train a natural language processing (NLP) model to derive labels from radiology text reports and then assign these labels to the corresponding MRI examinations. Recently, this technique has been demonstrated for labelling head computed tomography (CT) [6], chest CT [7], and chest radiograph [8, 9] examinations. A limitation of these studies is that performance was assessed by comparing labels derived from radiology reports by the model with reference-standard labels derived by manual inspection of the same radiology reports by radiologists. Ultimately, however, it is the agreement between predicted labels and the actual image findings which is most important for downstream computer vision training; in cases where radiology reports fail to capture the full gamut of findings (e.g. due to ‘satisfaction of search’ errors, or because the findings have been detailed in a previous report and not recapitulated in a follow-up report, e.g. ‘stable findings’ or ‘no interval change’), then this validation strategy may be insufficient.

In the context of head MRI examinations, NLP has been previously used to extract highly specific information from text reports, such as in quantifying the number of brain metastases from the reports of patients with brain metastasis [10], selecting MRI protocols [11], and highlighting acute strokes [12]. However, NLP has yet to be applied broadly to tasks such as labelling head MRI examinations in a manner suitable for general abnormality detection. This can be ascribed to the greater lexical complexity of MRI reports compared with other modalities such as CT, which is again due to the high soft-tissue contrast resolution of MRI which typically allows more detailed description of abnormalities and more refined diagnoses.

In the last 18 months, transformational developments within the field of NLP [13–17] have led to dramatic improvements in performance on a number of general [18] as well as more specialised [19, 20] language tasks. The purpose of our study was to build on these recent breakthroughs to create a state-of-the-art NLP model to automate the labelling of large MRI neuroradiology imaging datasets which could be used for downstream training of deep learning computer vision models to produce abnormality detection systems. We also sought to rigorously test our model by comparing labels predicted on the basis of radiology reports with labels generated via manual inspection of the corresponding images by a team of expert neuroradiologists. Given the growing evidence that significant discrepancies can exist between labels derived from radiology reports and those derived by radiologists interrogating the actual images [21, 22], determining the validity of using report labels as proxies for image labels in the context of head MRI examinations was an important aspect of our study.

## Methods

### Data

The UK’s National Health Research Authority and Research Ethics Committee approved this retrospective study. Radiology reports were extracted from the Computerised Radiology Information System (CRIS) (Wellbeing Software). Images were extracted from the Patient Archive and Communication Systems (PACS) workstations (Sectra). All data was de-identified. Reader image analysis was performed on PACS.

**Fig. 1:**
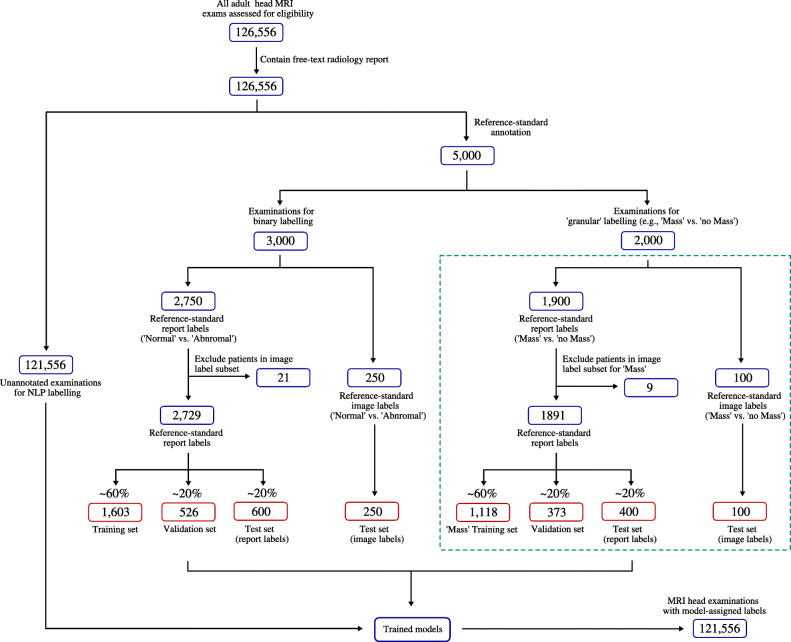
Flowchart showing datasets used to train, validate, and test our models. For each model, a subset of reports was assigned ‘reference-standard image labels’ (*n* = 250 for normal/abnormal, *n* = 100 for each of the 7 specialised categories) which served as a fixed hold-out ‘image label’ test set. After removing reports describing separate studies of patients in the test set, the remaining reports with ‘reference-standard report labels’ (e.g. *n* = 2729 for normal/abnormal, 1891 for ‘mass’/’no mass’ etc.) were split at the patient level into training and validation datasets, as well as a ‘report label’ test dataset, and model testing was performed in two ways: using the test set with (i) reference-standard report labels and (ii) reference-standard image labels. This splitting procedure was repeated 10 times for each category to generate model confidence intervals (the test set with reference-standard image labels always remained fixed). Note that the splitting procedure in the dashed teal box was performed separately for each of the 7 specialised categories of abnormality; however, only a single category (‘mass’) has been included for brevity. The full flow chart for all granular categories is available in the supplemental material (Fig. S1)

All 126,556 adult (≥ 18 years old) head MRI examinations performed at the King’s College Hospital NHS Foundation Trust between 2008 and 2019 were included in this study (Fig. 1). The corresponding 126,556 radiology text reports produced by 17 expert neuroradiologists (UK consultant grade; US attending equivalent) were also obtained. The neuroradiologists had different reporting styles. These reports were largely unstructured and typically comprised 5–10 sentences of image interpretation. Sometimes the reports included information from the MRI examination protocol, comments regarding the patient’s clinical history, and recommended actions for the referring doctor. The reports had often been transcribed using voice recognition software. We used type-token ratio and Yules I [23] to calculate the linguistic complexity of our report corpus, and compared this to similar-sized head CT [6] and chest radiograph [24] corpora from the radiology literature. Because differences in reporting styles could plausibly lead to poor model performance when classifying reports from an external hospital (‘domain shift’), 500 radiology reports from Guy’s and St Thomas’ NHS Foundation Trust were also obtained and used for additional model testing.

### Reference-standard report annotation

A subset of the reports was selected for annotation by 6 expert neuroradiologists (UK consultant grade; US attending equivalent). Five hundred reports were randomly sampled each year to create a 5000 report corpus for model training and evaluation. Prior to report labelling in this study, a complete set of clinically relevant categories of neuroradiological abnormality and a set of rules by which reports were to be labelled were developed (supplemental material). Fleiss’ kappa [25] was used to measure interrater reliability. All labelling was performed using a dedicated tool which we make openly available at https://github.com/MIDIconsortium/RadReports.

Three thousand reports were independently labelled by two neuroradiologists for the presence or absence of any abnormality. The level of the initial agreement between these two labellers was recorded, and where there was disagreement, a consensus classification decision was made with a third neuroradiologist. Separately, 2000 reports were independently labelled by three neuroradiologists for the presence or absence of 7 specialised categories of abnormality (i.e. 7 binary labels were assigned to each of these reports). These were acute stroke, mass, atrophy, vascular abnormality, small vessel disease [26], white matter inflammation, and encephalomalacia. The level of the initial agreement between these three labellers was recorded, with a consensus classification decision made with a fourth neuroradiologist where there was disagreement. We refer to the ‘presence or absence of any abnormality’ dataset as the ‘binary’ dataset and the ‘specialised categories of abnormality’ dataset as the ‘granular’ dataset.

### Reference-standard image annotation

In order to generate ‘reference-standard image labels’ for model testing, 950 head MRI examinations were randomly selected from the 5000 examinations with reference-standard report labels. Two neuroradiologists labelled 250 examinations as normal or abnormal applying the same framework used for report labelling—but interrogating the actual images. Separately, 7 datasets of 100 examinations were each labelled for the presence or absence of one of the 7 specialised categories. Each of these 7 datasets contained approximately 50 examinations with the specialised category of interest, and 50 examinations without (Fig. 1, Fig. S1). Creating balanced test datasets overcame underlying variations in prevalence for different categories, facilitating a fair comparison between each classifier.

All available sequences within a head MRI examination were interrogated when generating reference-standard image labels. This is consistent with the methodology for deriving labels from reports, as each report summarises the findings from all available sequences.

### NLP model generalisability

In order to determine the generalisability of our normal/abnormal classifier to radiology reports from an external hospital, 500 reports from Guy’s and St Thomas’ NHS Foundation Trust were also labelled by two neuroradiologists for the presence or absence of any abnormality, applying the same framework used to label reports from the King’s College Hospital NHS Foundation Trust. Again, a consensus classification decision was made with a third neuroradiologist where there was disagreement.

### Report pre-processing

Only those pre-processing steps required by transformer-based language models were performed [27]. Briefly, all reports were converted to lower case, and each report was converted into a list of unique integer token identifiers.

### Modelling

**Fig. 2:**
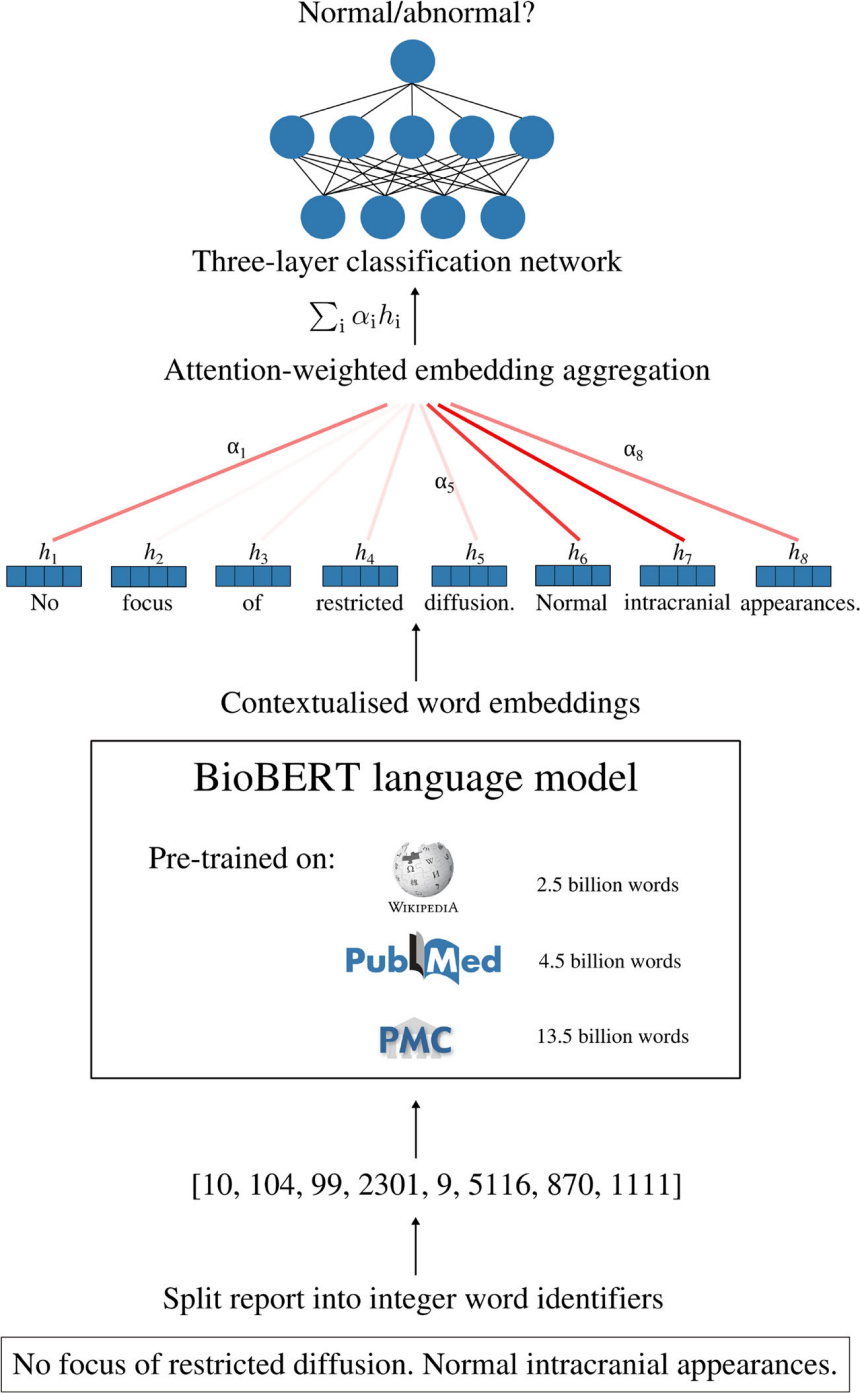
Deep learning MRI neuroradiology report classifier for automated dataset labelling. Free-text reports are converted into a list of integer word identifiers which are passed into a transformer-based language encoder network. This network converts each word into a 768-dimensional contextualised embedding vector and contains ~110 million parameters which are initialised with weights from BioBERT—a biomedical language model pre-trained on all of English Wikipedia (2.5 billion words), PubMed abstracts (4.5 billion words), and PMC full-text articles (13.5 billion words). A custom attention network aggregates these vectors into a report representation by a taking weighted sum of embedding vectors, with the weight of each word determined by its importance to the classification decision. A fully connected neural network with a single hidden layer then converts this to a probability that the report describes a category of interest, e.g. abnormal, mass, acute stroke. The entire network—i.e. the transformer language model, attention module, and classification network—is trained end-to-end on the basis of the binary cross-entropy between the model predictions and reference-standard report labels using the Adam optimizer

Our report classifier is built on top of BioBERT [19], a language model pre-trained on large-scale biomedical corpora which converts text tokens into contextualised vector representations suitable for downstream language processing tasks. We adapted BioBERT for report classification by adding a custom attention module which aggregates individual word vectors into a fixed-dimensional representation for each report, as well as a fully connected neural network with a single hidden layer which takes this vector representation as its input and outputs the probability that a report describes a given category of abnormality (Fig. 2). Further architectural details are provided in [28]. In total, 8 models were trained—one for normal/abnormal classification, and one for each of the 7 specialised categories of abnormality.

For each classifier, the corresponding dataset with reference-standard image labels (*n* = 250 examinations for binary classifier, *n* = 100 examinations for each of the 7 specialised classifiers) was put to one side for use as a hold-out test set. The remaining datasets of examinations with reference-standard report labels (after excluding patients appearing in the image label test set) were then randomly split into training (60%), validation (20%), and testing (20%) datasets. This split was performed at the patient level in order to prevent ‘data leakage’. Our overall approach to dataset generation is presented in Fig. 1, with a detailed description in the supplemental material. For each split, model checkpoints were saved after each epoch, and the model with the lowest validation loss was used for evaluation on (i) the test set with reference standard report labels, and (ii) the test set with reference-standard image labels. Following [29], we set the learning rate to 1e-5 in order to avoid ‘catastrophic forgetting’ of weights learned during pre-training; likewise, we set the batch size to 16 as this was the maximum possible size for a 12-GB graphics processing unit (GPU) and previous studies have shown that larger batch sizes give the best performance when fine-tuning BERT-based models [30]. Following [18], Adam optimizer was used to update model weights. All statistical analysis and modelling were performed using PyTorch 1.4.0, an open-source python-based scientific computing package which provides GPU acceleration for deep learning research [31]. The area under the receiver operating characteristic curve (AUC-ROC) was used to quantify model performance. To generate performance confidence intervals, the splitting procedure was repeated 10 times for each classifier. Accuracy, sensitivity, specificity, and F1 score were also calculated. Given the absence of a dedicated head MRI examination report classifier in the literature, to allow model comparison, we compared our model to the state-of-the-art head CT report classifier [6] using code available at https://github.com/aisinai/rad-report-anotator. The classifier is based on word2vec embeddings [32] and requires pre-training—for this, we used the remaining 121,556 reports (i.e. those not assigned reference standard labels). DeLong’s test [33] was used to determine the statistical significance of AUC-ROC values for different classifiers and for different evaluation procedures (i.e. reference-standard image labels and reference-standard report labels).

We applied t-distributed stochastic neighbour embedding (t-SNE) [34] to generate two-dimensional visualisations of the report embeddings used by our classifier and compared these to representations generated from word2vec embeddings. We also inspected the weights of our model’s custom attention layer to interrogate classification decisions, in particular erroneous decisions.

Code to enable readers to replicate these methods using their own datasets is available at https://github.com/MIDIconsortium/HeadMRIDatasetLabelling.

## Results

### Comparative lexical analysis

The lexical complexity of the MRI head report corpus was greater than similar-sized head CT [6] and chest radiograph [24] corpora (Table 1). Our dataset contained a higher number of unique words, both in absolute number (205,048) and per report (1.62), than these two other corpora, which is reflected in a higher Yule I and type-token-ratio score.

**Table 1:** Complexity analysis of head MRI, head CT [6], and chest radiograph [24] reports

Dataset	Number of reports	Total size of corpus (words)	Total number of unique words	Yule I	Type-token-ratio
Head MRI	126,556	14,183,182	205,048	79	0.019
Head CT [6]	96,303	12,110,849	145,257	34	0.011
Chest radiograph [24]	160,861	2,432,099	6481	29	0.002

### Reference-standard report annotation

Reference-standard report labels, along with the initial interrater agreement, for the two datasets are shown in Table 2. Across all abnormal reports in the granular dataset, the mean number of specialised abnormal labels per report was 1.56 (maximum = 5; mode = 1). The initial discrepancies between expert neuroradiologists using the same set of clear categorisation rules put into context the challenges facing an algorithm.

**Table 2:** Reference-standard report labels across all abnormality categories. We refer to the ‘presence or absence of any abnormality’ dataset as the ‘binary’ dataset and the ‘specialised categories of abnormality’ dataset as the ‘granular’ dataset. Granular definitions are provided in the supplemental material. Briefly, ‘small vessel disease’ refers to the presence of moderate or severe small vessel disease [26]; ‘vascular’ includes abnormalities such as aneurysms; ‘atrophy’ refers to volume loss in excess of age; ‘encephalomalacia’ refers to any cause of permanent tissue damage including previous surgery or the chronic sequelae of infarcts or haemorrhages

Dataset	Binary label dataset King’s College Hospital NHS Foundation Trust (*n* = 3000)	Binary label dataset Guy’s and St Thomas’ NHS Foundation Trust (*n* = 500)	Granular label dataset Guy’s and St Thomas’ NHS Foundation Trust (*n* = 2000)						
Category	Abnormal	Abnormal	Small vessel disease	Acute stroke	Mass	Vascular	White matter inflammation	Atrophy	Encephalomalacia
Number of examinations	1152	215	266	251	351	287	257	264	384
Interrater agreement (Fleiss kappa)	0.87	0.89	0.85	0.84	0.92	0.91	0.94	0.79	0.83

### NLP modelling

**Fig. 3:**
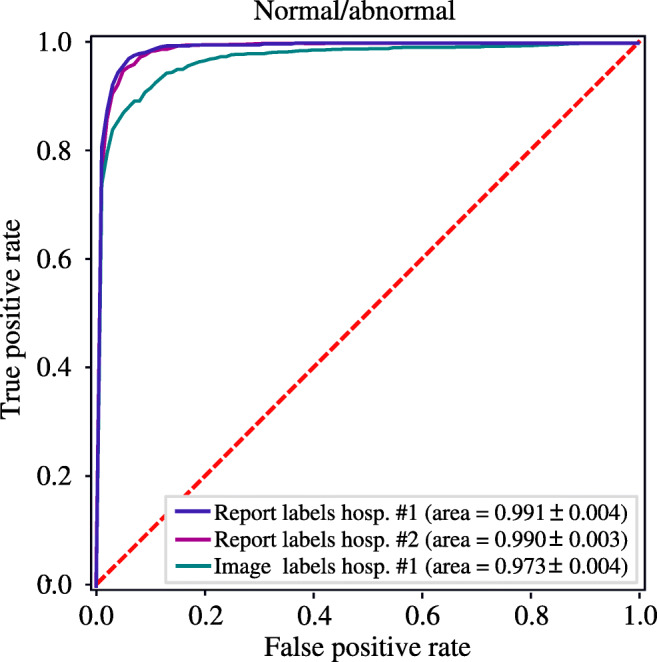
Receiver operating characteristic curve for the binary classifier evaluated on the reference-standard report label (indigo, *n* = 600) and reference-standard image label (teal, *n* = 250) test sets from the King’s College Hospital NHS Foundation Trust, and the reference-standard report label test set from Guy’s and St Thomas’ NHS Foundation Trust (magenta, *n* = 500). The area under the receiver operating characteristic curves and the corresponding 95% confidence intervals are also provided

Accurate neuroradiology report classification (AUC-ROC = 0.991) was achieved for the binary (i.e. normal or abnormal) classifier when tested against reference-standard report labels (Fig. 3, Table 3). Importantly, only a small reduction in performance (Δ AUC-ROC = 0.014) was seen when the classifier was tested against reference-standard image labels instead of reference-standard report labels (*p* < 0.05) (Fig. 3); in both cases, sensitivity and specificity of > 90% were achieved (Table 3). The model was generalised to reports obtained from Guy’s and St Thomas’ NHS Foundation Trust (∆AUC = 0.001) (Fig. 3). The model also outperformed (*p* < 0.05) a logistic regression model based on mean word2vec embeddings which is the state-of-the-art method for head CT report classification [6].

**Table 3:** Binary classifier performance evaluated on reference-standard report labels and reference-standard image labels. Comparison is made with a logistic regression model using mean word2vec embeddings and N-grams (*N* = 1, 2, 3) which has previously been shown to accurately classify head CT reports [6]. AUC-ROC, accuracy, sensitivity, specificity, and F1 score are provided, along with the corresponding 95% confidence intervals

Model	AUC-ROC	Balanced accuracy (%)	Sensitivity (%)	Specificity (%)	F1 (%)
Our model					
Report label test set (*n* = 600)	0.991 ± 0.004	95.9 ± 0.2	96.5 ± 0.1	95.3 ± 0.2	96.2 ± 0.2
Image label test set (*n* = 250)	0.973 ± 0.004	91.8 ± 0.6	91.4 ± 0.3	92.1 ± 0.5	93.0 ± 0.5
Word2vec model [6]					
Report label test set (*n* = 600)	0.969 ± 0.003	90.1 ± 0.3	89.1 ± 0.2	91.0 ± 0.2	90.3 ± 0.2
Image label test set (*n* = 250)	0.935 ± 0.004	86.2 ± 0.6	85.1 ± 0.4	87.3 ± 0.5	85.9 ± 0.5

**Fig. 4:**
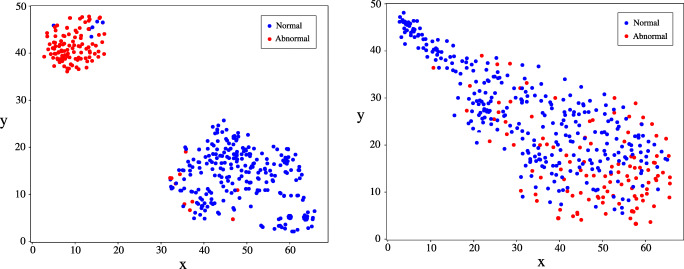
Two-dimensional visualisations of test set report embeddings generated by our model (left), and from mean word2vec embeddings (right), along with reference-standard report labels (abnormal: red, normal: blue). Representative examples of false-positive and false-negative misclassification are demonstrated in Fig. 5

**Fig. 5:**
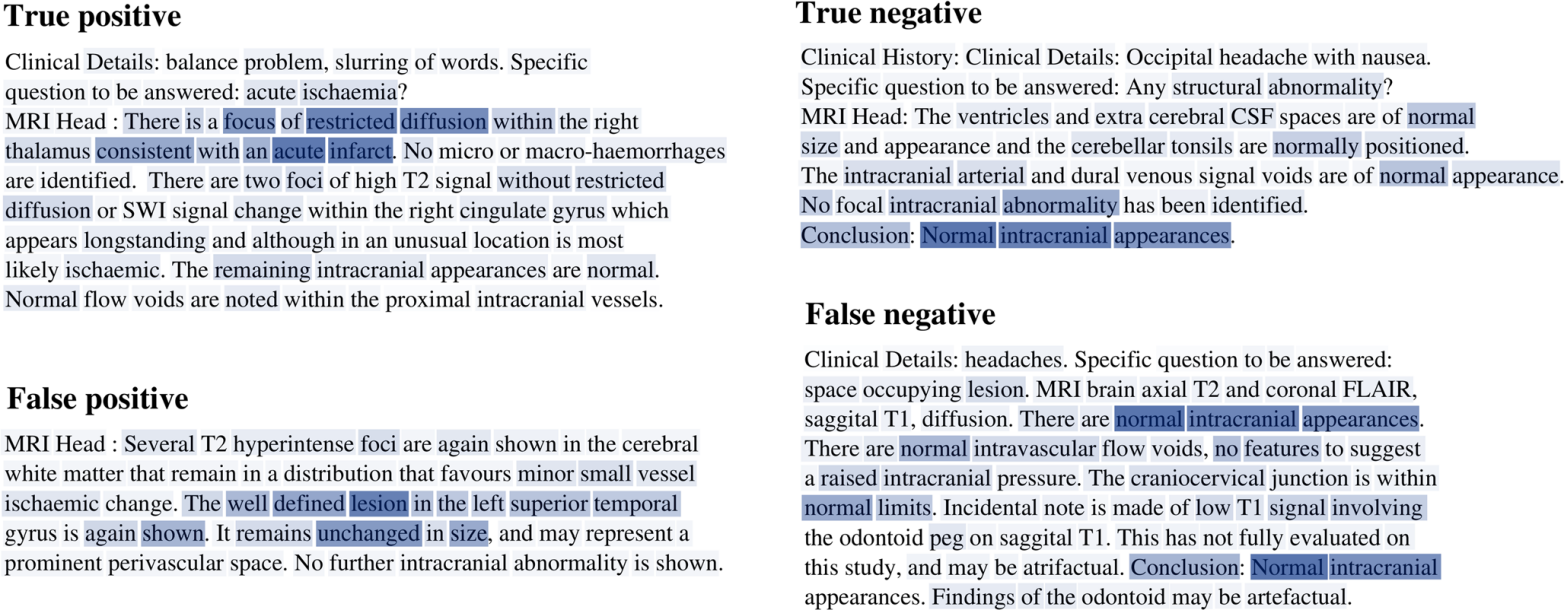
Visualisation of word-level attention weights including representative examples of false positive and false negative misclassification. Darker colour represents a higher contribution to the report representation used by the model for report classification. In **a** (true positive classification), the model assigned high weighting to several words in the sentence describing a ‘focus of restricted diffusion…consistent with an acute infarct’. In **b** (true negative classification), the model assigned the highest weighting to the words ‘normal’, ‘Intracranial’, and ‘appearances’. In **c** (false positive), the highest weighting was assigned to words describing a ‘well defined lesion’ which ‘remains unchanged in size’. However, this report was marked by our team of neuroradiologists as normal due to the likelihood that it represents a prominent perivascular space, a finding which our team consider normal unless excessively large. In **d** (false negative), the highest weighting was assigned to several instances of the phrase ‘normal intracranial appearances’. This example highlights a case where the neuroradiologist who reported the original scan reasonably deemed a finding insignificant—and used language accordingly—whereas our labelling team, in order to be as sensitive as possible, marked this report as abnormal. These representative examples demonstrate how our labelling framework errs towards the safest clinical decision. Additional examples of erroneous classification are available in the supplemental material

Using t-SNE, two-dimensional visualisations of the report representations used by the binary model were generated (Fig. 4). A clear clustering of normal and abnormal reports is seen, indicating that the model has separated the underlying factors of variation between these classes. In contrast, representations formed using mean word2vec embeddings exhibit considerably more overlap between the two classes. The relative importance of different words to the construction of each report representation can be determined by inspecting the weights of the attention network, providing a form of model interpretability (Fig. 5).

**Fig. 6:**
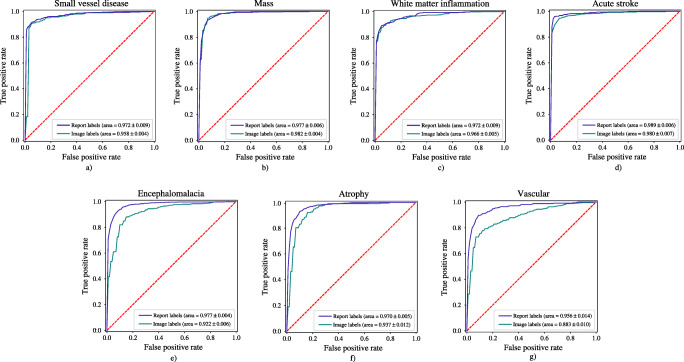
Receiver operator characteristic (ROC) curves for small vessel disease (**a**), mass (**b**), white matter inflammation (**c**), and acute stroke (**d**), encephalomalacia (**e**), atrophy (**f**), and vascular (**g**) classifiers evaluated on reference-standard report label (indigo, *n =* 400) and reference-standard image label (teal, *n* = 100) test sets. The area under the receiver operating characteristic curves and the corresponding 95% confidence intervals are also provided

For all granular abnormality categories studied, accurate neuroradiology report classification was achieved (AUC-ROC > 0.95, reference-standard report labels). For 4 of the 7 categories (acute stroke, mass, small vessel disease, and white matter inflammation), only a small reduction in performance (Δ AUC-ROC < 0.02) was observed when tested against reference-standard image labels instead of reference-standard report labels (*p* < 0.05) (Fig. 6a–d). For these categories, sensitivity and specificity of > 90% were achieved (Table 4). Interestingly, a larger drop in AUC-ROC was observed for atrophy (Δ AUC-ROC 0.037), encephalomalacia (Δ AUC-ROC 0.055), and vascular (Δ AUC-ROC 0.067) categories when tested against reference-standard image labels (*p* < 0.05) (Fig. 6e–g), highlighting discrepancies between labels derived from historical radiology reports, and those derived by manually scrutinising the images.

**Table 4:** Classifier performance for granular categories evaluated on reference-standard report and reference-standard image label test sets. AUC-ROC, accuracy, sensitivity, and specificity are provided, along with the corresponding 95% confidence intervals. Note that F1 was not included as this is not a suitable metric to compare model performance on datasets containing different degrees of class imbalance

		AUC-ROC	Balanced accuracy (%)	Sensitivity (%)	Specificity (%)
Report label test set (*n* = 400)	Mass	0.977 ± 0.006	93.1 ± 0.4	93.3 ± 0.4	92.8 ± 0.3
	Acute stroke	0.989 ± 0.006	96.0 ± 0.4	95.3 ± 0.3	96.6 ± 0.2
	Encephalomalacia	0.977 ± 0.004	91.2 ± 0.4	91.2 ± 0.6	91.3 ± 0.4
	Small vessel disease	0.972 ± 0.009	92.7 ± 0.9	91.7 ± 0.8	93.7 ± 0.3
	Atrophy	0.970 ± 0.005	90.1 ± 0.5	91.1 ± 0.4	89.1 ± 0.3
	Vascular	0.956 ± 0.014	89.3 ± 0.9	90.1 ± 0.7	88.4 ± 0.4
	White matter inflammation	0.972 ± 0.009	91.2 ± 0.6	90.1 ± 0.5	92.2 ± 0.4
Image label test set (*n* = 100)	Mass	0.982 ± 0.004	93.2 ± 1.4	94.3 ± 1.1	92.1 ± 0.8
	Acute stroke	0.980 ± 0.007	94.1 ± 0.8	93.8 ± 0.8	94.3 ± 0.2
	Encephalomalacia	0.922 ± 0.006	86.0 ± 1.0	85.9 ± 0.7	86.1 ± 0.7
	Small vessel disease	0.958 ± 0.004	91.2 ± 1.5	90.2 ± 0.9	92.2 ± 1.2
	Atrophy	0.937 ± 0.012	85.8 ± 1.8	85.0 ± 1.2	86.6 ± 1.4
	Vascular	0.883 ± 0.010	81.8 ± 2.6	81.1 ± 1.3	82.5 ± 2.3
	White matter inflammation	0.966 ± 0.005	90.7 ± 1.1	90.4 ± 0.6	90.9 ± 0.9

Once trained, our classifiers can be used to automatically assign labels to head MRI examinations by fixing the parameter weights and running each model in inference mode, thereby completing the final stage of a pipeline for labelling large datasets of head MRI examinations. To demonstrate feasibility, we assigned labels to the remaining 121,556 head MRI examinations that had not been used for reference-standard labelling (Fig. S3); this was achieved in under 30 min.

## Discussion

Artificial neural networks typically require tens of thousands of labelled images to achieve the best possible performance in image recognition tasks. This represents a bottleneck to the development of deep learning systems for complex image datasets, particularly MRI which is fundamental to neurological abnormality detection. In this work, we have developed a dedicated neuroradiology report classifier which can automate image labelling by deriving labels from radiology reports and accurately assign important labels to the corresponding MRI examinations. It was feasible for our model to assign more than 100,000 MRI scans as normal or abnormal—as well as allocating specialised labels to abnormal scans—in under 30 min, a task that would likely take years to complete manually.

Our study builds on recent transformational developments in NLP, culminating with the introduction of the Bidirectional Encoder Representations from Transformers (BERT) model and the biomedical variant BioBERT. Both of these models were pre-trained on huge collections of text—BioBERT, for example, was trained on English Wikipedia and all PubMed Central abstracts and full-text articles, totalling more than 20 billion words—meaning that considerable low-level language comprehension can be inherited by initialising downstream networks with weights from these parent models, so that fewer labelled examples are necessary for model training.

Additionally, BERT and BioBERT provide contextualised word embeddings. Before 2018, state-of-the-art document classification models used pre-trained word2vec or GloVe [35] embeddings. However, a fundamental limitation is that these embeddings are context-independent. For example, the vector for the word ‘stroke’ would be the same when present in the sentence ‘restricted diffusion consistent with acute stroke’ as it would be in the sentence ‘no features suggestive of acute stroke’. Context independence is particularly problematic for complex, unstructured, reports like those in our MRI corpus as these often include descriptions, proceeded by distant negation, of abnormalities which are not present, including those that are being searched for in light of the clinical information.

Previous studies have only reported model performance on a hold-out set of labelled reports [6, 7, 9], and to date, there has been no investigation into the general validity of NLP-derived labels for head MRI examinations [36]. An important question that we investigated in this study was the validity of using report labels as proxies for image labels. By comparing our model’s predictions with reference-standard image labels derived by our team of neuroradiologists on the basis of manual inspection of 950 images, we have shown that binary labels indicating the presence or absence of any abnormality can reliably be assigned using our NLP model. We have also shown that labels for four specialised categories of abnormality (mass, small vessel disease, white matter inflammation, and acute stroke) can be accurately assigned.

Whilst label information was accurately extracted from the original reports for all categories (AUC > 0.95, reference-standard report label validation), the original reports less accurately represented the actual image findings for three categories of abnormality (encephalomalacia, vascular, and atrophy), as evidenced by the greater ΔAUC-ROC. This represents a source of error unrelated to NLP model performance (a text classifier cannot detect findings which are not reported). There may be several reasons for this discrepancy. First, in the presence of more clinically important findings, neuroradiologists often omit descriptions of less critical abnormalities which may not necessarily change the overall conclusion or instigate a change in the patient’s management. For example, we noted that MRI reports were often insensitive to non-critical findings such as micro-haemorrhages (vascular category) or minor parenchymal residua from an intraventricular drain tract (encephalomalacia). A second source of low sensitivity is the observation that radiology reports are often tailored to specific clinical contexts and the referrer. A report aimed at a neurologist referrer who is specifically enquiring about a neurodegenerative process in a patient with new-onset dementia, for example, may make comments about subtle parenchymal atrophy. In contrast, parenchymal volumes may not be scrutinised as closely in the context of a patient who has presented with a vascular abnormality, such as an aneurysm, and the report is aimed at a vascular neurosurgeon. The drop in accuracy for these three categories highlights an important and novel contribution of our work, namely that validation against manual inspection of radiology examinations by experienced radiologists may be necessary to rigorously determine the validity of using report labels as proxies for image labels.

Although our neuroradiology report classifiers are highly accurate, they are not perfect models (i.e. they achieve AUC < 1, Fig. 3). This will result in some small fraction of images being mislabelled. Recent studies have shown that this ‘label noise’ can impact the performance of deep learning models [37, 38]. Nonetheless, the level of label noise which results from using our models is modest and is in fact below known error rates present in commonly-used computer vision datasets (e.g. ImageNet, which is estimated to have label noise as high as 10% [39, 40]); as such, minimal impact on downstream computer vision performance can be expected.

A limitation of our work is that our sample training cohort may not be representative of every neurological patient population. However, the sample was large, and obtained from a sizeable hospital and university cluster where imaging is obtained for all neurological, neurosurgical and psychiatric disorders, and also included healthy volunteers. This hospital department also consists of 17 expert neuroradiologists with different reporting styles. Furthermore, our normal/abnormal classifier demonstrated minimal degradation in performance when applied to reports from an external hospital. Together, this would suggest that our study findings are reasonably representative of large hospitals catering for a wide range of neurological abnormalities reported by neuroradiologists. Nonetheless, as part of future work, we plan to further investigate the generalisability of our classifiers to examinations from other hospitals.

In conclusion, we have developed an accurate neuroradiology report classifier to automate the labelling of head MRI examinations. Assigning binary labels (i.e. normal or abnormal) to images from reports alone is highly accurate. In contrast to the binary labels, the accuracy of more granular labelling is dependent on the category. Our model performed the labelling task in a small fraction of the time it would take to perform manually. Together, these results overcome a critical bottleneck to the development and widespread translation of deep learning computer vision systems for image recognition tasks in radiology.

## Supplementary information

ESM 1 (DOCX 1086 kb)

## Abbreviations

AUC-ROC: Area under the receiver operating characteristic curve
BERT: Bidirectional Encoder Representations from Transformers
BioBERT: Bidirectional Encoder Representations from Transformers for Biomedical Text Mining
GloVe: Global Vectors for Word Representation
NLP: Natural language processing
t-SNE: t-distributed stochastic neighbour embedding

## Declarations

### Guarantor

The scientific guarantor of this publication is Thomas C. Booth.

### Statistics and biometry

No complex statistical methods were necessary for this paper, but DW and JC have considerable statistical experience.

### Methodology

• retrospective

• experimental

• multicentre study
