# Supplementary material for: Deep learning to automate the labelling of head MRI datasets for computer vision applications
Source: Eur Radiol. 2021 Jul 20;32(1):725–36. doi: 10.1007/s00330-021-08132-0 (PMC8660736; doi:10.1007/s00330-021-08132-0)
Supplement: Supplementary file 1 — (DOCX 1086 kb) [file 330_2021_8132_MOESM1_ESM.docx]

**Supplemental material**

**Figures**

**
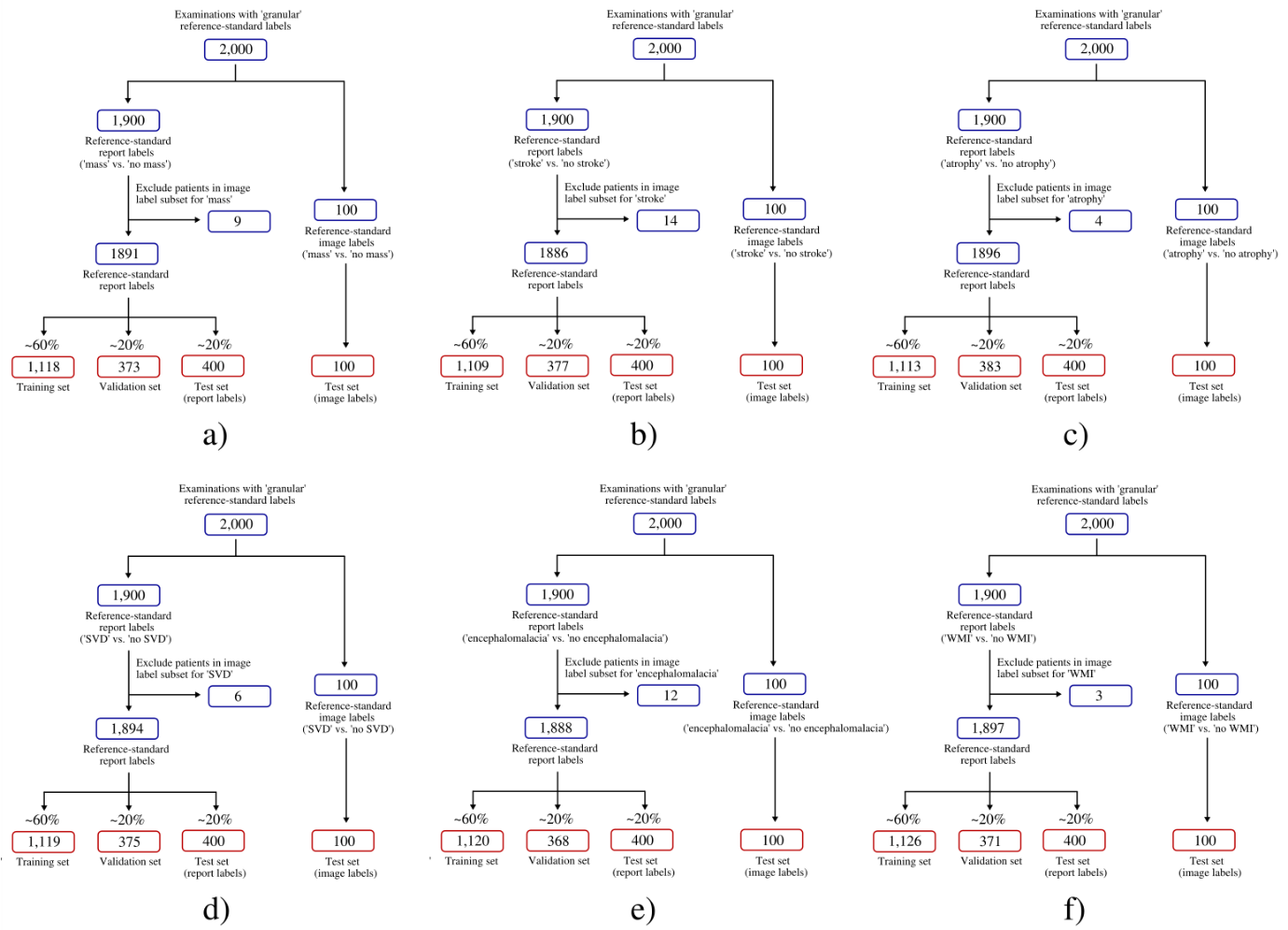
**

**Figure S1:** Flow chart showing datasets used to train, validate, and test our ‘granular’ models. For each model, a subset of reports was assigned ‘reference-standard image labels’ (n=100) which served as a fixed hold-out ‘image label’ test set. After removing reports describing separate studies of patients in the test set, the remaining reports with ‘reference-standard report labels’ (1891 for ‘mass’/’no mass’ etc.) were split at the patient level into training and validation datasets, as well as a ‘report label’ test dataset, and model testing was performed in two ways: using the test set with (i) reference-standard report labels; and (ii) reference-standard image labels. This splitting procedure was repeated 10 times for each category to generate model confidence intervals (the test set with reference-standard image labels always remained fixed). Note that WMI and SVD refer to white matter inflammation and small vessel disease, respectively.


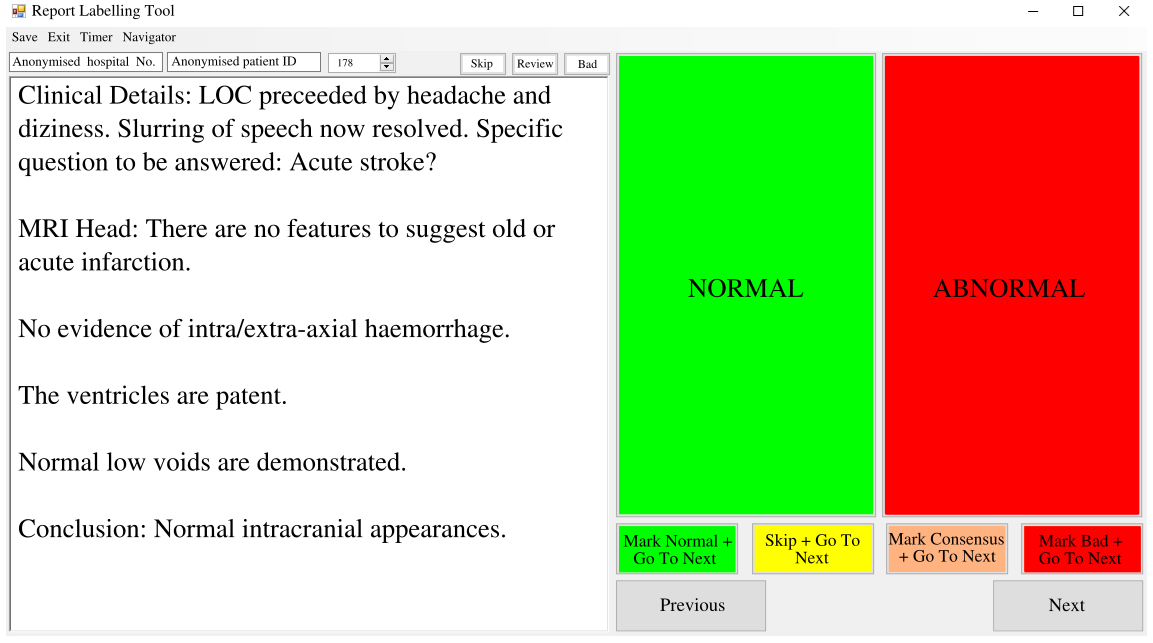


**Figure S2**: Binary report labelling tool for annotating neuroradiology reports. Each report is automatically loaded from a comma separated variable (CSV) or Excel (Microsoft) file. Users read the report and label by clicking the appropriate button. Reports can also be marked as requiring a consensus decision if the user is unsure. All labels are written to an output file, and labelling can be paused and then resumed from the last saved checkpoint. The example report should be marked as normal. Note that the report is largely unstructured and there are typographical and voice recognition errors in the clinical details provided by the referring clinician and the report produced by the neuroradiolgist, respectively.

**
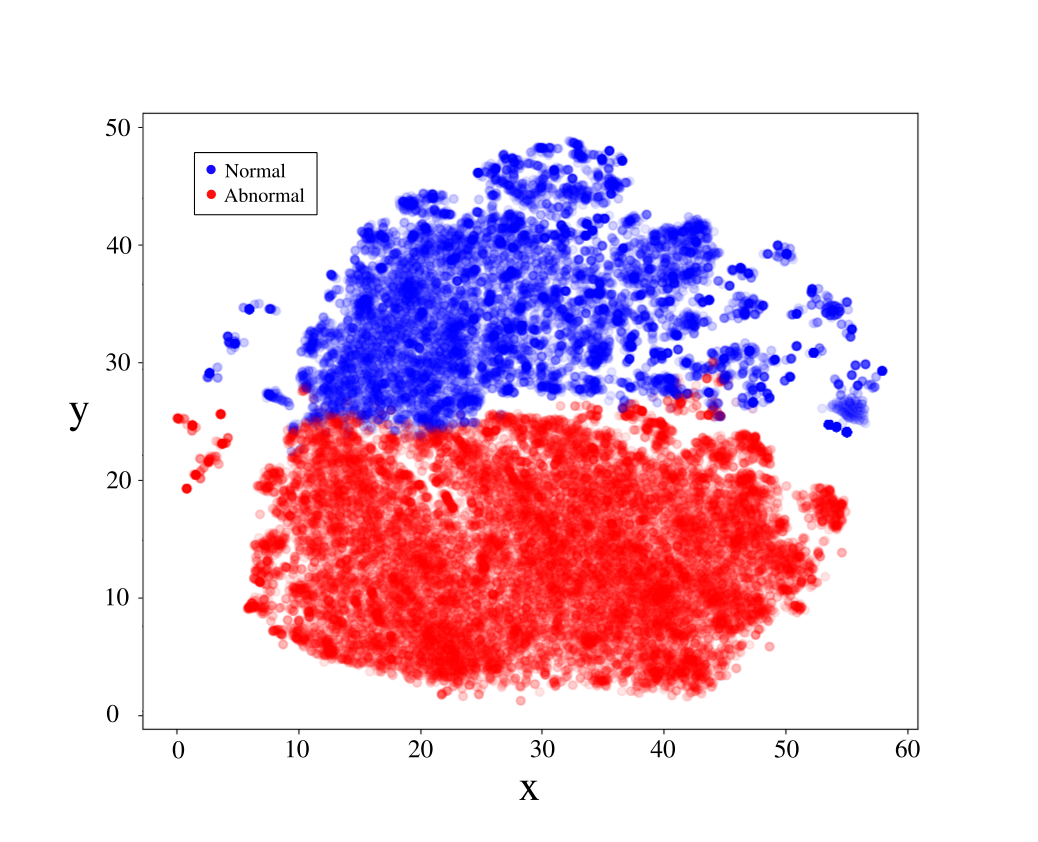
**

**Figure S3:** t-SNE visualisation of vector embeddings and binary labels assigned to the remaining 121,556 head MRI examinations that had not been used for reference standard labelling. This was completed in under 30 minutes demonstrating feasibility of the final stage of a pipeline for labelling large datasets of head MRI examinations.

**
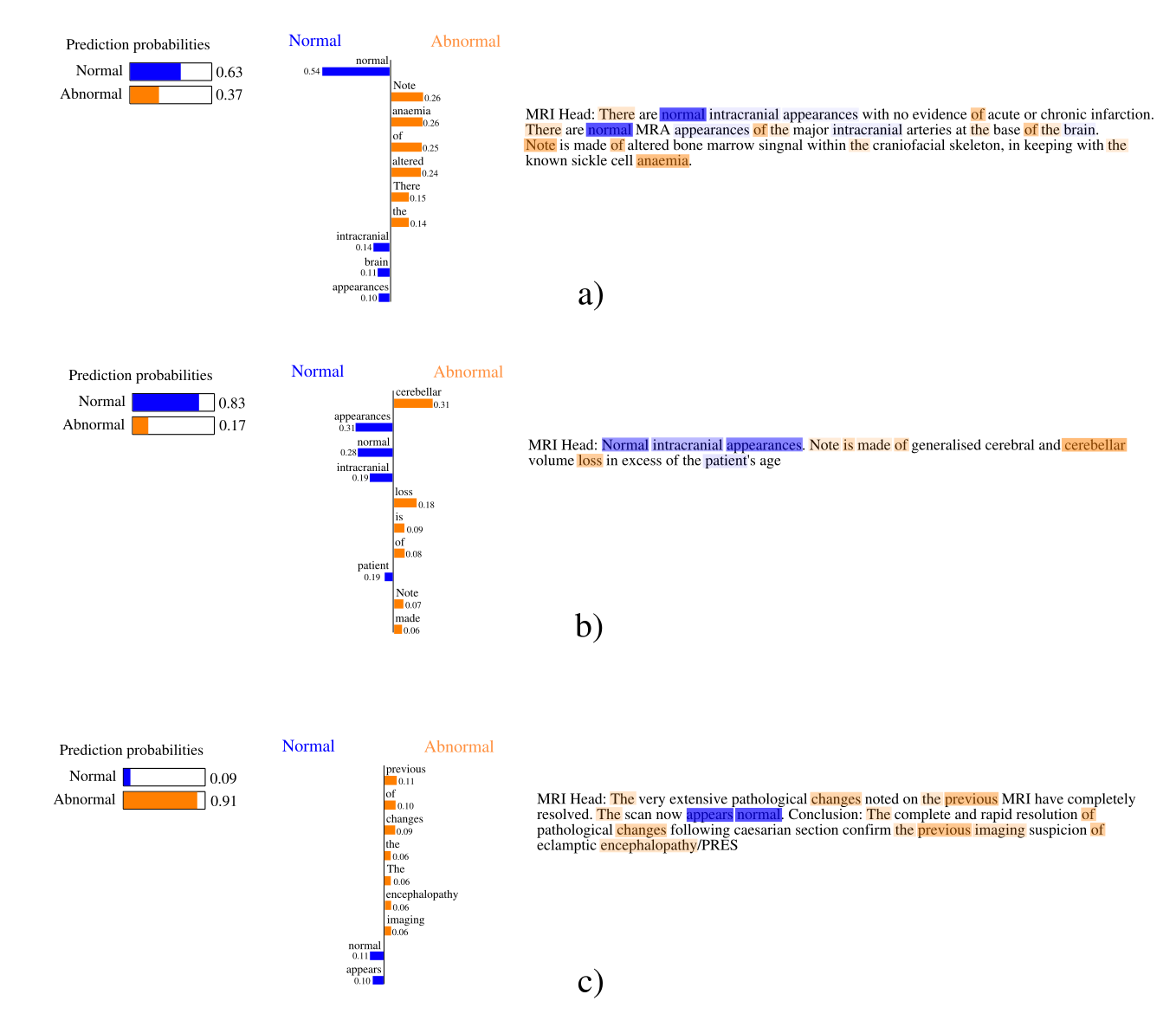
**

**Figure S4:** Examples of false positive and false negative classifications. Salient words are determined using LIME [1], a model-agnostic method for explaining machine learning predictions. The magnitudes of the word importance weights agree with those derived by an attention-based analysis e.g., as shown in Fig. 5 in the main manuscript. These examples are representative; our model sometimes makes mistakes when the reporting radiologist concludes that there are ‘normal intracranial appearances’, but also identifies a minor incidental finding (a) and (b). These examples highlight cases where the neuroradiologist who reported the original scan reasonably deemed a finding insignificant - and used language accordingly - whereas our labelling team, in order to be as sensitive as possible, marked this report as abnormal. Likewise, our model sometimes made mistakes when reports described the complete resolution of findings seen in previous studies (c); in these cases, the words and phrases used to describe the resolution of findings are often similar to that used to diagnose the same findings (e.g., ‘confirm the previous suspicion of eclamptic encephalopathy/PRES’).

**Dataset Description**

Our dataset consisted of all 126,556 head MRI examinations performed at King’s College Hospital NHS Foundation Trust between 2008 - 2019 (images + reports).

Five hundred reports were randomly sampled from each year to create a dataset of 5000 examinations for model training and evaluation. Of these 5000 examinations, 3000 were labelled for the presence or absence of any abnormality by manually scrutinising the corresponding radiology reports (‘reference-standard report labels’). A subset of examinations (n_examinations = 250 corresponding to n_patients = 250) were also assigned ‘reference-standard image labels’ by interrogating the actual images.

Separately, 2000 examinations were labelled for the presence or absence of 7 specialised categories of abnormality (acute stroke, mass, atrophy, vascular abnormality, small vessel disease, white matter inflammation, encephalomalacia) by manually scrutinising the corresponding radiology reports (‘reference-standard report labels’); a subset of these examinations (n_reports=700 corresponding to n_patients=700) were also assigned reference-standard image labels.

Each model was trained independently: we elected to train 8 models rather than a single model with 8 outputs to enable stratification of the training/validation/testing splits by abnormality frequency (i.e. achieving approximately equal fractions of positive and negative classes in each split).

For the binary classifier (normal/abnormal), the 250 reports with reference-standard image labels were separated prior to training and these served as the “reference-standard image label” test set (note that this set was fixed for all train/validation splits described below). Following this, a search of the remaining 2750 reports revealed that 21 reports described separate examinations for patients that were also in the n=250 image label test set. These were removed to prevent data leakage. The remaining 2729 reports were then randomly divided into training/validation/testing datasets; the test set here is the “reference-standard report label” test set. The split was performed at the patient level; again to prevent data leakage where there were separate examinations for the same patient. Note that the numbers given in Fig.1 are representative but are only exactly true for a single train/validation/test split, and could change by a tiny amount for each split (~1%-3%) due to the requirement of patient-level splitting i.e. it is not in general possible to get exactly 80%/20%/20% at the report level when the splitting is done at the patient level.

For each data split, the model was trained on the training dataset (report labels), the loss was monitored using the validation dataset (report labels), and then the model with the lowest loss on the validation set was used for testing. Testing was always performed in two ways for each split: (1) using the test dataset for that split with reference-standard report labels (n = 600 in the figure for the binary branch); and (2) using the fixed set of 250 reports with “reference-standard image labels”. This procedure was repeated 10 times to generate confidence intervals.

For the 7 specialised (“granular”) classifiers, we were again very careful to prevent data leakage (right hand branch in Fig. 1 in the manuscript).

The following “Venn diagram” shows how the 2000 reports were labelled:

First, all 2000 reports (large blue rectangle) were assigned 7 binary labels i.e., stroke: yes/no, vascular: yes/no, mass: yes/no, atrophy: yes/no, encephalomalacia: yes/no, SVD: yes/no, WMI: yes/no. Note that these reports could have more than one “positive” label e.g., ‘mass’ and ‘SVD’ etc.

Following this, 7 sets of 100 examinations were randomly selected from these 2000 examinations (using up-sampling – see below) and assigned “reference-standard image labels” (coloured circles). Note that there is no overlap in the granular image label test sets i.e., a given report appears in at most one image label test set (hence there are no intersections between circles in the Venn diagram). For each category, the splitting procedure was analogous to that of the binary classifier. Therefore, the split was performed at the patient level; again to prevent data leakage where there were separate examinations for the same patient. A test set (n=100) with image labels was separated from the larger dataset with report labels and a small number of reports were excluded from the remaining 1900 reports due to patient overlap (in the figure, n_excluded=9 for ‘mass’, but this is slightly different for each category, see Fig. S1). Training/validation/testing splits in the remaing 1891 reports were generated at the patient level; the test set here is the “reference-standard report label” test set. Models were tested (1) using the test dataset for that split with reference-standard report labels (n = 400 in the figure); and (2) using the fixed set of 100 reports with “reference-standard image labels (again image label test set remained fixed, and report label test sets differed for each of the 10 splits). This is summarised in the two additional Venn diagrams below. Note that the white circles indicate reports that have been excluded from the dataset with report labels for training/validating/testing in order to prevent data leakage.


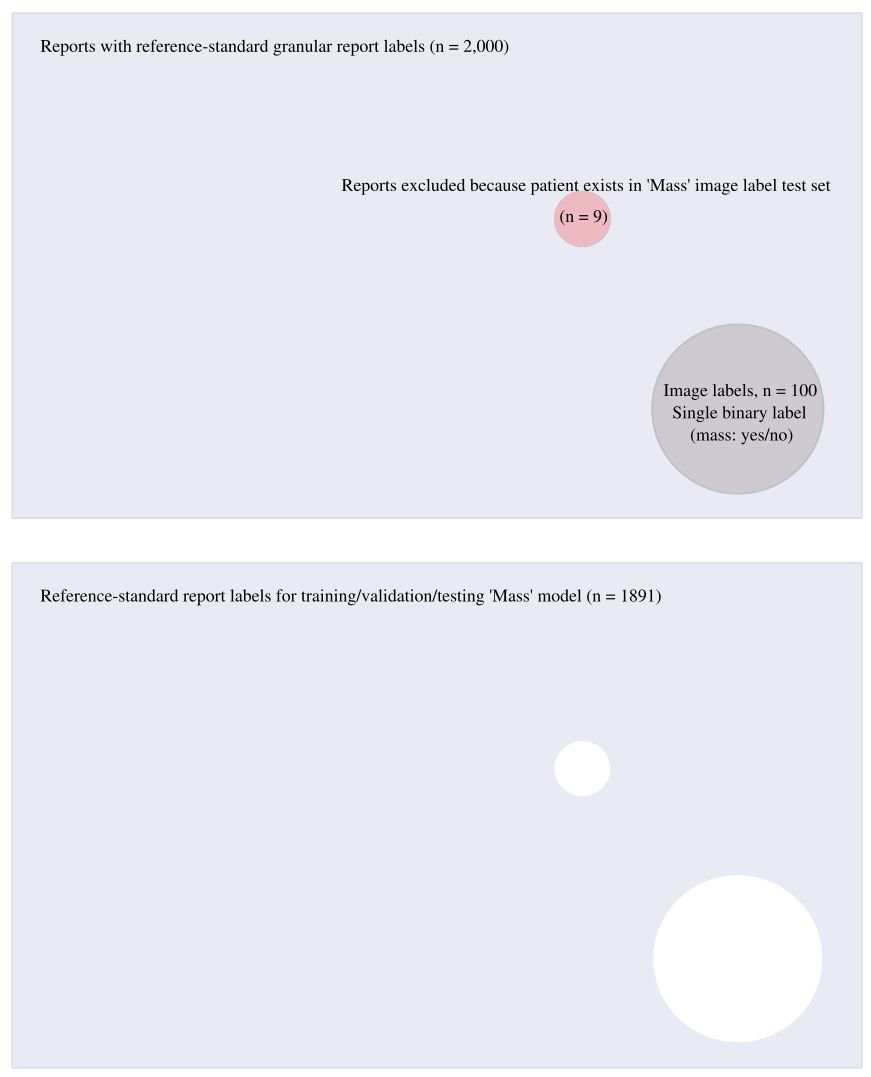


**Ontology**

Pre-existing ontologies were first explored [2] but constraints led us to a design our own. For example, ICD-10 Version:2019 [3] the latest version of the *International Statistical Classification of Diseases and Related Health Problems* contains many relevant neuroradiology disease classification codes due to complications or manifestations of a disorder that can lead to brain abnormalities for example, human immunodeficiency virus (HIV) encephalopathy or a brain metastasis from melanoma. However, multiple abnormalities are included in: I – XIV (A to N), XVII (Q) XX (S,T), XXI (V, X, Y). Brain abnormalities are frequently found in VI G00-G99 Diseases of the Nervous System e.g. G00 meningitis. Many are seen in V F00-F09 Mental and Behavioural Disorders e.g. F00 dementia in Alzheimer disease. Even XV (O) Pregnancy, Childbirth and the Puerperium e.g. O11 pre-eclampsia, can involve brain abnormalities. The breadth of codes, the paucity of relevant hierarchical structure, and the difficulty linking disease classification to head MRI examination findings, meant that this was not fit for use as an ontology for head MRI abnormality detection. The Systematized Nomenclature of Medicine—Clinical Terms (SNOMED CT) [4], and variants thereof, have a broader classification and the medical concepts through which it is organized are linked together in logical connections to create aggregate concepts or conditions. Nonetheless, experiments showed this was not fit for purpose for neuroradiology tasks. RadLex is a radiological lexicon, the principle aim of which is to develop a standardized vocabulary for radiologists [5]. The “imaging observations” are not linked in a pertinent hierarchy, however, and do not contain important findings seen on head MRI examinations such as “mass” or similar, and there a few “parent” terms with “children” terms. There is no “MRI” or “neuroradiology” specific ontology from which a hierarchy flows.

As these systems were not designed for our use case, we developed our own fit-for-purpose ontology. This was based on capturing most common conditions with a limited number of categories. The limited number of categories ensured that manual labelling was feasible, classification was feasible and application was practical. Abnormalities were grouped together into a category if they demonstrated (1) similarity on MRI e.g., mass morphology is broadly similar whether the mass is a meningioma or abscess, and (2) histopathological similarity e.g., arteriovenous malformation and developmental venous anomaly are similar. In the latter example the MRI appearance of vascular morphology and signal characteristics are also similar. Assignment to groups required judicious compromise because abnormalities may not have both MRI and histopathological similarity.

Our team designed a complete set of categories containing clinically relevant abnormalities thereby accurately capturing the full range of pathologies (and normal variants mimicking pathologies) which present on head MRI examinations. The aim here was to try and emulate the behaviour of a neuroradiologist in the real world, whilst ensuring that findings that may generate a downstream clinical intervention for an abnormal finding are included. In our ontology, clinical intervention includes referral for case discussion at a multi-disciplinary team meeting. In some cases, for example the discovery of a developmental venous anomaly, the referring clinician may not understand the clinical relevance of the reported finding and may thus need to request clarification of clinical relevance at a multi-disciplinary team meeting. By erring on the side of sensitivity our labelling framework will lead to an algorithm producing the safest clinical decision (Fig. 4).

The development process required the inspection > 1000 radiology reports by our team of experienced neuroradiologists before an exhaustive and consistent set of abnormality categories, as well as the rules by which reports were to be labelled, could be finalised. The rules and definitions recursively evolved during the course of the practice labelling experiments. Once developed, however, these remained fixed for all experiments and results presented in the current work.

To allow other researchers to bypass this step and accelerate their research, we make our refined abnormality definitions and labelling rules available below. This may also encourage standardization across research groups.

**Labelling tool**

Our manual labelling campaign was considerably aided by our development of a dedicated labelling ‘app'. This easy-to-use app allows clear report visualisation and allows labelling of reports through a graphical user interface (GUI), and includes functionality for flagging difficult cases for group consensus/review. Two apps were developed - one for binary labelling (Fig. S2), and one for more granular labelling - and we make both available to other researchers at https://github.com/MIDIconsortium/RadReports.

**Binary abnormality definitions**

Abnormal is defined as one or more abnormality described below.

Normal is defined as no abnormality described below.

**Granular (specialised) abnormality definitions**

**Small vessel disease**

Reference [6] gives a classification system for white matter lesions (WMLs) summarised as:

1. Mild - punctate WMLS: Fazekas I

2. Moderate - confluent WMLs: Fazekas II

3. Severe - extensive confluent WMLs: Fazekas III

To create a binary categorical variable from this system, if the report is described as “unsure”, “normal” or “mild” this is categorized as normal as this never requires treatment for cardiovascular risk factors. However, if there is a description of moderate or severe WMLs, the report is categorized as abnormal as these cases sometimes require treatment for cardiovascular risk factors.

Included as normal are descriptions of scattered non-specific “white matter dots” or “foci of signal abnormality” (unless a more defuse or specific pathology is implied) and small vessel disease described as “minor”, “minimal” or “modest”.

Conversely, those cases which are described as “mild to moderate”, “confluent”, or “beginning to confluence” small vessel disease are treated as abnormal.

Genetic small vessel disease, in particular Cerebral Autosomal Dominant Arteriopathy with Subcortical Infarcts and Leukoencephalopathy (CADASIL), is considered abnormal.

**Mass**

All the following intracranial masses are categorized as abnormal:

− Neoplasms (tumours)

- Intra-axial including all primary and secondary neoplasms
- Extra-axial including all primary and secondary neoplasms
  - Pituitary adenomas included
- Lipomas included

− Tumour debulking or partial resection as this implies residual tumour (note: these are labelled as both “encephalomalacia“ and “mass“ abnormalities)

− Ependymal, subependymal or local meningeal enhancement (non-surgical) in the context of a history of an aggressive infiltrative tumour

− Abscess

− Cysts

- Retrocebellar cyst (mega cisterna magna not included)
- Arachnoid cysts
- Pineal cysts and choroid fissure cysts
- Rathke cleft cysts

− Focal cortical dysplasia, nodular grey matter heterotopia, subependymal nodules and subcortical tubers

− Chronic subdural haematoma or hygroma (i.e. cerebrospinal fluid (CSF) equivalent)

− Perivascular spaces normal unless giant

– MRI examinations for stereotactic surgical planning alone may have very brief reports. In these scenarios it is typically evident from the clinical information provided that there is a mass e.g., surgical planning for glioblastoma.

Note that findings that typically may have minimal clinical relevance when confirmed by a neuroradiology expert, are included in this category e.g., arachnoid cyst. The rationale is that such a finding might generate a referral to a multidisciplinary team meeting for clarification clinical relevance. We consider that a referral to a multidisciplinary team meeting is a clinical intervention and we aim to ensure that any findings that generate a downstream clinical intervention are included.

**Vascular**

All the following are categorized as abnormal for vascular:

− Aneurysm

• including coiled aneurysms regardless of whether there is a residual neck or not

− Arteriovenous malformation

− Arteriovenous dural fistula

− Cavernoma

− Capillary telangiectasia

− Chronic / non-specific microhaemorrhages

− Petechial haemorrhage

− Developmental venous anomaly

− Venous sinus thrombosis

– Vasculitis if associated with vessel changes such as luminal stenosis or vessel wall enhancement

− Arterial occlusion / flow void abnormality or absence

− Venous sinus tumour invasion (this is labelled as both “vascular“ and “mass“ abnormalities)

− Arterial stenosis. If constitutional / normal variant not included.

− Vascular-like findings which are considered normal include descriptions of sluggish flow, flow-related signal abnormalities (unless they raise the suspicion of thrombus) and vascular fenestrations.

Note that findings that typically may have minimal clinical relevance when confirmed by a neuroradiology expert, are included in this category e.g., developmental venous anomaly. The rationale is that such a finding might generate a referral to a multidisciplinary team meeting for clarification of clinical relevance. We consider that a referral to a multidisciplinary team meeting is a clinical intervention and we aim to ensure that any findings that generate a downstream clinical intervention are included.

**Encephalomalacia**

All the following are categorized as abnormal for encephalomalacia:

− Gliosis

− Encephalomalacia

− Cavity

− Post-operative tissue changes / appearances are included as encephalomalacia

− Tumour debulking or partial resection as this implies residual tumour (note: these are labelled as both “encephalomalacia“ and “mass’“ abnormalities)

− Chronic infarct / sequelae of infarct

− Chronic haemorrhage / sequelae of haemorrhage (with / without haemosiderin staining)

− Cortical laminar necrosis

Encephalomalacia-like findings which are considered normal unless there is a clear description of related parenchymal injury include craniotomy, burr-holes, posterior fossa decompression, and 3rd ventriculostomy

**Acute stroke**

All the following are categorized as abnormal for acute stroke:

− Acute / subacute infarct (if demonstrating restricted diffusion)

• Include if there are other descriptors indicating a subacute nature such as swelling

even though restricted diffusion has normalised

– If a single ischaemic event with both diffusion restricting and non-restricting elements then this is labelled as an “acute stroke” abnormality (rather than an “encephalomalacia” abnormality)

− Parenchymal post-operative restricted diffusion secondary to retraction injury

– Mitochondrial Encephalopathy with Lactic Acidosis and Stroke-like episodes (MELAS) if associated with restricted diffusion

– Hypoxic ischaemic injury if associated with restricted diffusion

– Vasculitis if associated with acute / subacute infarct

– “Mature”, “established”, “chronic” or “old” infarcts without other descriptors are labelled as “encephalomalacia” abnormalities

**White matter inflammation**

All the following are categorized as abnormal for white matter inflammation:

– Multiple sclerosis (MS) including when some plaques show cavitation (low T_1_ signal)

– Other demyelinating lesions including Acute Disseminated Encephalomyelitis (ADEM) and Neuromyelitis Optica spectrum disorder (NMO)

– Inflammatory lesions in Radiologically Isolated Syndrome / Clinically Isolated Syndrome

– Focal cortical thinning i.e., secondary to chronic subcortical / cortical lesions, are labelled as “encephalomalacia“ abnormalities

– Progressive Multifocal Leukoencephalopathy (PML)/ Immune Reconstitution Inflammatory Syndrome (IRIS)

– Leukoencephalopathies - congenital or acquired (including toxic)

– Encephalitis / encephalopathy if it involves the white matter, e.g. related to human immunodeficiency virus (HIV) and congenital cytomegalovirus (CMV)

– Posterior Reversible Encephalopathy Syndrome (PRES)

– Osmotic demyelination (central pontine myelinolysis/ extrapontine myelinolysis)

– Susac syndrome

– Radiation if describing white matter abnormality

– White matter changes in the context of vasculitis if clearly attributed to vasculitis.

– Amyloid-related inflammatory change / inflammatory

**Atrophy**

Volume loss in excess of age

**General abnormality category**

In addition to these 7 specialised categories, there is a generalised “abnormal“ category. This includes reports describing any abnormality from the 7 granular categories, as well as any of the following:

Hydrocephalus:

– Acute

– Trapped ventricle

– Chronic / stable / improving hydrocephalus (it does not matter whether its compensated or not)

– Ventricular enlargement

– normal pressure hydrocephalus (NPH)

Haemorrhage:

– Any acute / subacute haemorrhage parenchymal, subarachnoid, subdural, extradural

– Acute microhaemorrhages / petechial haemorrhages

Foreign body:

– Shunts

– Clips

– Coils

– If significant metalwork is involved in skull repair e.g. in a cranioplasty (or the occasional craniotomy causing extreme intracranial MRI signal distortion)

– If craniotomies are not causing anything other than slight artefact, then these are considered normal

Extracranial:

– Total mastoid opacification / middle ear effusions

– Complete opacification / obstruction of the paranasal sinuses

– Mucosal thickening is not included

– If there is clearly a well-defined unambiguous polyp then label as abnormal.

– If “retention cysts“ or “polypoid mucosal thickening“ then label as normal. If it is something indistinguishable which could be a retention cyst / polyp then label as normal.

– Anything leading to sinus obstruction always label as abnormal.

– Calvarial / extra-calvarial masses

– Osteo-dural defects

– Encephaloceles

– Pseudomeningoceles

– Extracranial vascular abnormalities i.e., below the petrous segment e.g. cervical internal carotid artery (ICA) dissection

– Extracranial masses including lipoma or sebaceous cyst

– Orbital abnormalities

• Including optic nerve pathology affecting the orbital segment of the nerve i.e., meningioma

– Cases with isolated tortuous optic nerve sheath complexes with no other features suggestive of raised intracranial pressure, are labelled as normal

– Eye prostheses and proptosis

– Pseudophakia is labelled as normal

– Bone abnormality e.g., low bone signal secondary to haemoglobinopathy

– Basilar invagination

– Hyperostosis is considered normal

– Thornwald cysts are considered normal

Intracranial miscellaneous:

– Cerebellar ectopia

– Brain herniation (e.g., through a craniectomy defect)

– Clear evidence of intracranial hypertension (e.g. prominent optic nerve sheaths AND intrasellar subarchnoid herniation)

• Isolated intrasellar subarachnoid herniation / empty sella is labelled normal

• Isolated tapering of dural venous sinuses is labelled normal

– Clear evidence of intracranial hypotension (e.g., pituitary enlargement AND pachymeningeal thickening)

• If subdural collections present, these are also labelled as “mass“

– Cerebral oedema or reduced CSF spaces from parenchymal swelling

– Absent or hypoplastic structures such as agenesis of the corpus callosum

– Meningeal thickening or enhancement for example in the context of neurosarcoid or vasculitis

– Enhancing or thickened cranial nerves

– Infective processes primarily involving the meninges or ependyma (i.e. ventriculitis or meningitis)

– Encephalitis if primarily involving the cortex (herpes simplex virus (HSV)/ autoimmune encephalitis)

– Excessive or unexpected basal ganglia or parenchymal calcification

– Optic neuritis involving the intracranial segments of the optic nerves or chiasmitis

– Adhesions / webs

– Pneumocephalus

– Colpocephaly

– Superficial siderosis

– Ulegyria

– Focal areas of signal intensity (FASIs) / Unidentified bright objects (UBO)

– Basal ganglia / thalamic changes in the context of metabolic abnormalities

– Neurovascular conflict fulfilling conditions of nerve distortion AND nerve root entry zone involvement

– Band heterotopia and polymicrogyria

– Hypophysitis

– Seizure related changes

– Amyotrophic lateral sclerosis (ALS).

**Impact of label “noise”**

Although our neuroradiology report classifiers are highly accurate, they are not perfect models (i.e. they achieve AUC < 1, Fig. 3). This will result in some small fraction (e.g., 5 - 10 % for ‘normal’/’abnormal’) of images being mislabelled. Recent studies have shown that “label noise” can impact the performance of deep learning models. Nonetheless, the level of label noise which results from using our models is modest, and is in fact below known error rates present in commonly-used computer vision datasets e.g. ImageNet; as such, minimal impact on downstream computer vision performance can be expected.

Nonetheless, our rigorous image label validation approach can in fact help make the downstream computer vision model more tolerant of mislabelled images than it might otherwise be. This is because many state-of-the-art approaches to noise-correction involve representing label noise through a “transition matrix” which specifies the class-conditional probability that an image has been mislabelled. In general, this matrix is unknown *a priori* and must be learned as part of model training, although this often results in the model erroneously learning an “identity function” for the transition matrix, with the result that noise correction fails [7]. However, as part of model evaluation we naturally create a “confusion matrix” which, by specifying the probability of false positives, false negatives etc., is precisely equal to the transition matrix, meaning that state-of-the-art noise correction strategies can be applied “out of the box”.

**Supplemental material references:**

1. Ribeiro, M. T., Singh, S., & Guestrin, C. (2016). " Why should I trust you?" Explaining the predictions of any classifier. In *Proceedings of the 22nd ACM SIGKDD international conference on knowledge discovery and data mining* (pp. 1135-1144).
2. Wang, K. C. (2018). Standard lexicons, coding systems and ontologies for interoperability and semantic computation in imaging. *Journal of digital imaging*, *31*(3), 353-360.
3. Steindel, S. J. (2010). International classification of diseases, clinical modification and procedure coding system: descriptive overview of the next generation HIPAA code sets. *Journal of the American Medical Informatics Association*, *17*(3), 274-282.
4. SNOWMED CT. International Health Terminology Standards Development Organisation. <http://www.ihtsdo.org/snomed-ct/>. Accessed July 31, 2015
5. Rubin, D. L. (2008). Creating and curating a terminology for radiology: ontology modeling and analysis. *Journal of digital imaging*, *21*(4), 355-362.
6. Fazekas, F., Chawluk, J. B., Alavi, A., Hurtig, H. I., & Zimmerman, R. A. (1987). MR signal abnormalities at 1.5 T in Alzheimer's dementia and normal aging. *American journal of roentgenology*, *149*(2), 351-356.
7. Sukhbaatar, S., Bruna, J., Paluri, M., Bourdev, L., & Fergus, R. (2014). Training convolutional networks with noisy labels. [3rd International Conference on Learning Representations, ICLR 2015](https://nyuscholars.nyu.edu/en/publications/training-convolutional-networks-with-noisy-labels) - San Diego, United States
